# Partial absence of PD‐1 expression by tumor‐infiltrating EBV‐specific CD8^+^ T cells in EBV‐driven lymphoepithelioma‐like carcinoma

**DOI:** 10.1002/cti2.1175

**Published:** 2020-09-09

**Authors:** Yannick Simoni, Etienne Becht, Shamin Li, Chiew Yee Loh, Joe Poh Sheng Yeong, Tony Kiat Hon Lim, Angela Takano, Daniel Shao Weng Tan, Evan W Newell

**Affiliations:** ^1^ Vaccine and Infectious Disease Division Fred Hutchinson Cancer Research Center Seattle WA USA; ^2^ Agency for Science, Technology and Research Singapore (A*STAR) Singapore Immunology Network (SIgN) Singapore; ^3^ ImmunoScape Pte Ltd Singapore; ^4^ Department of Anatomical Pathology Singapore General Hospital Singapore General Hospital Singapore; ^5^ Division of Medical Oncology National Cancer Centre Singapore (NCCS) Singapore; ^6^ Agency for Science, Technology and Research (A*STAR) Genome Institute of Singapore (GIS) Singapore; ^7^ Senior Corresponding Author

**Keywords:** cancer, CD39, CD8, EBV, LELC, lymphoepithelioma‐like carcinoma, PD‐1, tetramer, tumor

## Abstract

**Objectives:**

Lymphoepithelioma‐like carcinoma (LELC) is an uncommon lung cancer, typically observed in young, non‐smoking Asian populations. LELC is associated with Epstein–Barr virus (EBV) infection of lung tumor cells of epithelial origin, suggesting a carcinogenic role of EBV as observed in nasopharyngeal carcinoma (NPC). Here, we studied the antigen specificity and phenotype of EBV‐specific CD8^+^ T cells in blood and tumor of one LELC patient positive for EBV infection in lung tumor cells.

**Methods:**

Using multiplex MHC class I tetramers, mass cytometry and mRNA sequencing, we studied EBV‐specific CD8^+^ T cells at the transcriptomic and phenotypic levels in blood and tumor tissues of the LELC patient.

**Results:**

Lymphoepithelioma‐like carcinoma lung tumor cells were positive for EBV infection. In both blood and tumor tissues, we detected two populations of EBV‐specific CD8^+^ T cells targeting the EBV lytic cycle proteins: BRLF1 and BMLF1. Transcriptomic analyses of these two populations in the tumor, which can be considered as tumor‐specific, revealed their distinct exhausted profile and polyclonal TCR repertoire. High‐dimensional phenotypical analysis revealed the distinct phenotype of these cells between blood and tumor tissues. In tumor tissue, EBV‐specific CD8^+^ TILs were phenotypically heterogeneous, but consistently expressed CD39. Unexpectedly, although the LELC tumor cells expressed abundant PD‐L1, these tumor‐specific CD8^+^ tumor‐infiltrating lymphocytes (TILs) mostly did not express PD‐1.

**Conclusion:**

Epstein–Barr virus‐specific CD8^+^ TILs in EBV‐driven tumor are heterogeneous and partially lack PD‐1 expression, suggesting that anti‐PD1/PD‐L1 immunotherapy may not be an appropriate strategy for disinhibiting EBV‐specific cells in the treatment of LELC patients.

## Graphical abstract

We studied the antigen specificity and phenotype of CD8^+^ tumor‐infiltrating lymphocytes (TILs) in one LELC patient positive for EBV infection in lung tumor cells. Transcriptomic analyses of EBV‐specific CD8^+^ TILs reveal their distinct exhausted profiles and polyclonal TCR repertoire. Although the LELC tumor cells expressed abundant PD‐L1, EBV‐specific CD8^+^ TILs mostly did not express PD‐1, but express CD39.

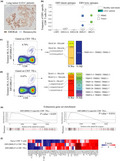

## Introduction

A better characterisation of tumor‐specific T cells is essential in order to improve efficacy of immune‐based therapies.^1^ However, because of the presence of cancer‐unrelated ‘bystander’ T cells in the tumor^2^, ^3^ and the difficulty to identify tumor‐specific T cells (e.g. neoantigen prediction),^4^ the study of these cells remains challenging in cancer. Human cancers associated with viral infections represent approximatively 10% of the worldwide cancer incidence.^5^ Because tumor cells present well‐characterised viral antigens through MHC class I molecule, investigating virus‐specific T cells in virus‐associated cancers is relevant to study tumor‐specific T cells in the tumors.

Oncogenic viruses such as hepatitis B virus (HBV) in liver cancer, human papillomavirus (HPV) in cervical cancer or Epstein–Barr virus (EBV) in B‐cell lymphoma disturb biological pathways to replicate in tumor‐infected cells and escape the immune surveillance.^6^, ^7^ Importantly, none of these viral infections on their own are sufficient to induce cancer but are closely related to genetic susceptibility (e.g. single nucleotide variant, driver mutation) and environmental factors (e.g. viral co‐infection, diets).^7^, ^8^, ^9^ Lymphoepithelioma‐like carcinoma (LELC) is a rare cancer, characterised by a massive infiltration of lymphocytes.^10^ The term LELC refers to its histological resemblance with the lymphoepithelioma tumor observed in nasopharyngeal carcinoma (NPC). The first case of LELC in the lung was reported in 1987^11^ and has been observed in several other tissues^12^ such as gastrointestinal tract,^13^ salivary glands,^14^ skin,^15^ breast^16^ or vagina.^17^ Because of the absence of driver mutation (e.g. EGFR, ALK) in the majority of patients with LELC of the lung,^18^ effective treatment options are limited (e.g. TKI drugs). A strong association between LELC in the lung and EBV infection has been observed in Asian populations.^19^ Based on the observations made for NPC, the mechanistic model suggests that EBV virus is not an initiating factor in the oncogenic process, but a tumor‐promoting agent. Loss of heterozygosity in epithelial cells associated with genetic (e.g. Asian ethnicity) and environmental factors (e.g. salt fish) is an early event in the pathology of the disease. Infection of these DNA‐damaged epithelial cells by EBV, followed by the expression of EBV latent genes, will provide growth and survival advantages to these cells (e.g. BCL2 overexpression) and lead to the development of carcinoma that may finally result in metastasis.^7^, ^9^ Study of EBV‐specific T cells in EBV‐associated tumors (i.e. NPC, LELC) represents an important step in developing more efficient therapeutic tools to treat these cancers, such as TCR‐engineered T cells targeting EBV epitopes presented by tumor cells, or improved checkpoint blockade immunotherapy (e.g. anti‐PD‐1, anti‐PD‐L1).

Here, using MHC class I tetramers to identify EBV‐specific CD8^+^ TILs in a patient with LELC in the lung, we report the simultaneous detection of two EBV‐specific populations. Although these two populations share common characteristics, we highlight the polyclonality of their T‐cell receptors, their heterogeneous exhausted/dysfunctional profile and the unexpected partial absence of PD‐1 surface protein expression.

## Results

### Identification of EBV‐specific CD8^+^ T cells in EBV‐driven LELC cancer

**Figure 1:**
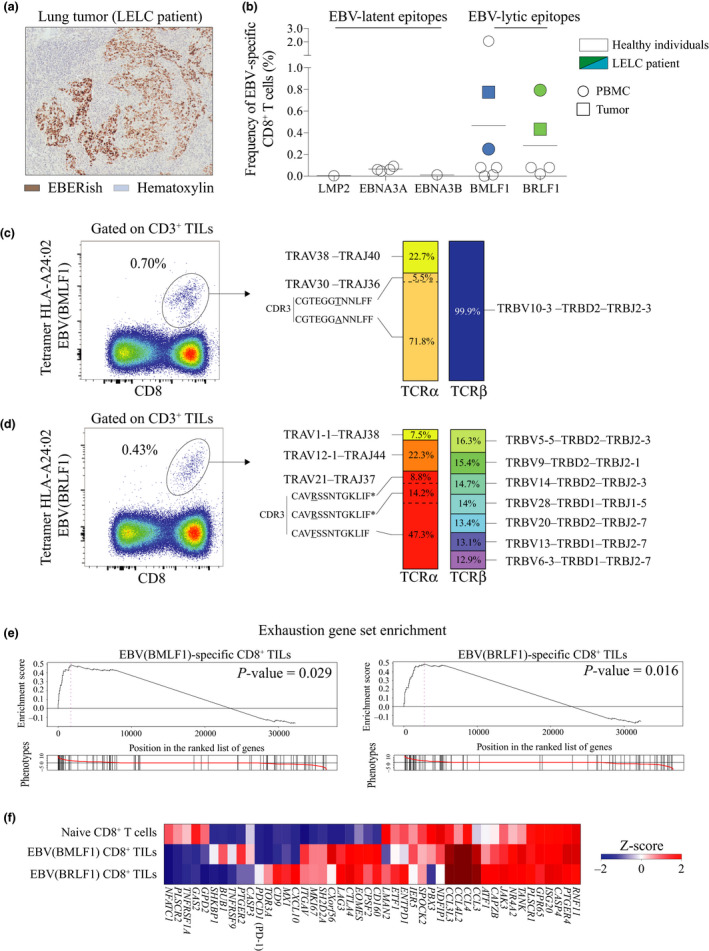
Identification of EBV‐specific CD8^+^ TILs in EBV‐infected LELC of the lung. **(a)** Immunohistochemistry staining of the lung tumor from LELC patient stained with haematoxylin (blue) and Epstein–Barr virus‐encoded small RNA in situ hybridisation (EBERish – brown) (patient A311). **(b)** Frequency of EBV‐specific CD8^+^ T cells for EBV lytic and EBV latent epitopes in HLA‐A*24: 02 LELC patient (*n* = 1) and healthy individuals (*n* = 7). **(c)** Flow staining of sorted EBV (BMLF1)‐specific CD8^+^ TILs using HLA‐A*24: 02 tetramer (DYNFVKQLF) (left panel). Frequency of tetramer‐positive cells among CD8^+^ TILs. TCRα and TCRβ clones’ repertoire of bulk EBV(BMLF1)‐specific CD8^+^ TILs (right panel). Data from LELC patient A311. **(d)** Flow staining of sorted EBV (BRLF1)‐specific CD8^+^ TILs using HLA‐A*24: 02 tetramer (TYPVLEEMF) (left panel). Frequency of tetramer‐positive cells among CD8^+^ TILs. TCRα and TCRβ clones’ repertoire of bulk EBV (BRLF1)‐specific CD8^+^ TILs (right panel), * the same CDR3 amino acid sequence but different nucleotide sequence. Data from LELC patient A311. **(e)** Enrichment of the gene set for exhausted T cells in naive CD8^+^ T cells from PBMC (flow‐sorted CCR7^–^ CD45RO^–^), and EBV (BMLF1)‐ or EBV (BRLF1)‐specific CD8^+^ TILs. Gene position on the left indicates enrichment in EBV (BMLF1)‐ or EBV (BRLF1)‐specific CD8^+^ TILs. Gene position on the right indicates enrichment in naive CD8^+^ T cells. Data from LELC patient A311. **(f)** Heat map of gene set for exhaustion shown in **e**. Data from LELC patient A311.

A 69‐year‐old Asian woman, non‐smoker, was diagnosed with a lung cancer. Histological examination from resected tumor of this patient indicated a lymphoepithelioma‐like carcinoma (LELC) structure with an abundant infiltration of immune cells. Because LELC of the lung is strongly associated with the presence of Epstein–Barr virus (EBV) in tumor cells,^19^ we performed EBV‐encoded RNA in situ hybridisation (EBERish) staining on tumor tissue sections. We confirmed the presence of EBV viral RNA in the tumor cells of this patient (Figure 1a). Based on the HLA‐A alleles expressed by this patient, we screened for EBV‐specific CD8^+^ T cells using HLA‐A*24: 02 tetramers loaded with different EBV epitopes (Supplementary figure 1a). We also included PBMC from healthy individuals in our experiment. We detected two EBV‐specific CD8^+^ T‐cell populations recognising BMLF1 (DYNFVKQLF) and BRLF1 (TYPVLEEMF) epitopes in both blood and tumor of this patient (Figure 1b and Supplementary figure 1a). Since EBV proteins are presented by MHC class I molecules from the tumor cells, EBV‐specific CD8^+^ TILs can be considered as tumor‐specific. Both epitopes identified derive from proteins involved in the early lytic cycle of EBV,^7^ strongly suggesting that EBV is producing virions in tumor cells. We did not detect any CD8^+^ T cells specific for antigens associated with EBV latent cycle in this patient despite the addition of epitopes derived from three latency‐associated gene products (LMP2, EBNA3A or EBNA3B) (Figure 1b, see Discussion).

### EBV‐specific CD8^+^ TILs in EBV‐driven LELC have a polyclonal TCR repertoire and are exhausted

We sorted by FACS and analysed the transcriptomic profile of both populations of tumor‐infiltrating EBV‐specific CD8^+^ T cells (TILs) in this patient. Of note, we could not perform this experiment using PBMC because of the low number of antigen‐specific T cells obtained after flow sorting. Sorted cells were analysed without prior expansion that could alter the TCR repertoire and transcriptomic profile. We first studied the TCR repertoire and observed that both populations of EBV‐specific CD8^+^ TILs were polyclonal for their TCRα with three and four different TRA clones detected for each epitope (Figure 1c and d). Interestingly, BMLF1 tetramer^+^ cells expressed only one TCRβ chain B clone (Figure 1c). In contrast, for BRLF1 tetramer^+^ cells, seven different TRB clones were detected at a similar frequency (Figure 1d). This result highlighted the TCR polyclonality of antigen‐specific CD8^+^ TILs. Because of this polyclonality, measuring skewed TCR repertoire in the tumor would not necessarily reflect an enrichment of tumor‐specific CD8^+^ TILs.

Gene set enrichment analysis revealed a significant exhausted profile for both populations of EBV‐specific CD8^+^ TILs (Figure 1e). Although both populations similarly expressed genes involved in dysfunction/exhaustion status (i.e. *CTLA4, LAG3, ENTPD1*) or proliferation (i.e. *MKI67*), we surprisingly observed a dichotomy for some other genes (Figure 1f). For example, the inhibitory receptor *PDCD1* (PD‐1) was only detected in EBV(BRLF1)‐specific CD8^+^ TILs at low level (Figure 1f). Despite being in the same tumor microenvironment and being specific for the same virus, the two populations showed a distinct exhaustion profile at the gene level (see Discussion). To confirm these differences, we further analysed surface marker expression by both populations at the single‐cell level using mass cytometry.

### EBV‐specific CD8^+^ TILs in EBV‐driven LELC are phenotypically heterogeneous and express CD39

**Figure 2:**
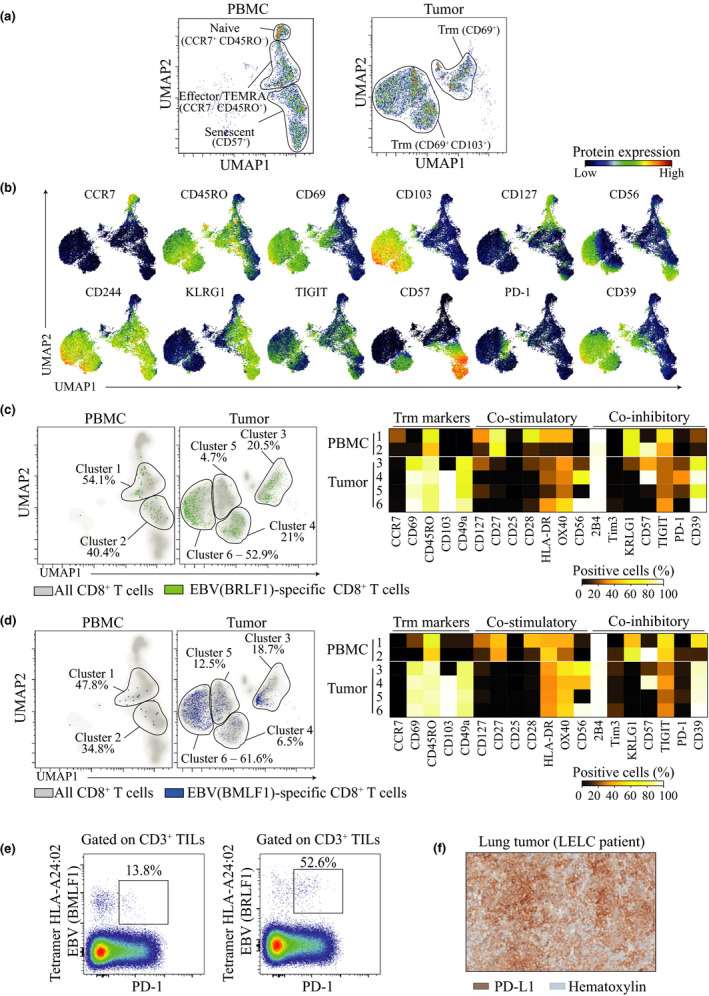
EBV‐specific CD8^+^ TILs in EBV‐infected LELC are heterogeneous and partially lack expression of PD‐1. **(a)** UMAP plots of CD8^+^ T cells from PBMC (left) and tumor tissue (right) isolated from LELC patient A311 and acquired by mass cytometry. **(b)** Concatenated peripheral and tumor‐infiltrating CD8^+^ T‐cell UMAP plot showing expression intensities for various phenotypic surface markers. **(c)** UMAP plots of EBV (BRLF1)‐specific CD8^+^ T cells (green) plotted on total CD8^+^ T cells (grey) in PBMC and tumor from LELC cancer patient (left panel). Based on the clusters identified for BRLF1‐specific CD8^+^ T cells, frequencies of cells positive for each surface marker were calculated and shown in a heat map (right panel). **(d)** UMAP plots of EBV (BMLF1)‐specific CD8^+^ T cells (blue) plotted on total CD8^+^ T cells (grey) in PBMC and tumor from LELC cancer patient (left panel). Based on the clusters identified for BMLF1‐specific CD8^+^ T cells, frequencies of cells positive for each surface marker were calculated and shown in a heat map (right panel). **(e)** Flow‐cytometry staining showing expression of PD‐1 by EBV (BMLF1)‐ and EBV (BRFL1)‐specific CD8^+^ TILs. Frequencies among tetramer^+^ CD8^+^ TILs. **(f)** Immunohistochemistry staining of the lung tumor from LELC patient stained with haematoxylin (blue) and PD‐L1 (brown) (patient A311).

We developed a mass‐cytometry panel of metal‐labelled antibodies to analyse CD8^+^ T cells from the LELC‐resected lung tumor and paired PBMC.^20^ This panel included a broad range of phenotypic markers that allowed to simultaneously measure the expression of markers associated with T‐cell differentiation, activation, tissue residency and dysfunction/exhaustion. For high‐dimensional analysis of tetramer^+^ CD8^+^ T cells, we used uniform manifold approximation and projection (UMAP), which accounts for non‐linear relationships between markers and projects high‐dimensional data into two dimensions (called UMAP1 and UMAP2) by making a pairwise comparison of cellular phenotypes to optimally plot similar cells nearby to each other.^21^, ^22^ We observed that CD8^+^ T cells derived from PBMC or tumor were localised in different areas of the UMAP plot, indicating non‐overlapping phenotypes of CD8^+^ T cells between the two tissues (Figure 2a). In PBMC, we observed clusters of CD8^+^ T cells that were identified with characteristics of naive (CCR7^+^ CD45RO^–^), effector (CCR7^–^ CD45RO^+^) or senescent (CD57^+^) cells (Figure 2a, b). In the tumor tissue, CD8^+^ TIL clusters expressed tissue‐resident memory (Trm markers) (CD69^+^ CD103^+/–^) (Figure 2b). When plotting both populations of EBV‐specific CD8^+^ T cells on the UMAP plot, we observed the presence of two different clusters in PBMC (clusters 1 and 2) and four in tumor tissue (clusters 3 to 6), highlighting their phenotypic heterogeneity (Figure 2c, d). Nevertheless, both populations of EBV‐specific CD8^+^ TILs shared several similar characteristics. For instance, both of them displayed an effector phenotype (CCR7^–^ CD45RO^+^) and expressed Trm markers (CD69, CD49a). Interestingly, EBV‐specific CD8^+^ TILs expressed CD39 – a marker reported to be induced after chronic antigen stimulation – but not PD‐1 (Figure 2c, d). The heterogeneity of both EBV‐specific CD8^+^ TILs was mainly driven by differential expression of integrin CD103 (cluster 3), the senescence marker CD57 (cluster 4) or the absence of adhesion molecule CD56 (cluster 5). Altogether, these observations showed the heterogeneity of the two EBV‐specific CD8^+^ TIL populations at the protein level in the same tumor microenvironment.

### Expression of PD‐1 is partially absent on EBV‐specific CD8^+^ TILs from the LELC patient

Expression of the inhibitory receptor PD‐1 is known to inhibit tumor‐specific CD8^+^ T‐cell activation.^23^, ^24^ Treatment of cancer patients with anti‐PD‐1 or anti‐PD‐L1 has successfully induced tumor regression in some cases. However, anti‐PD‐1 treatment efficacy varies across patients and cancer types.^25^ Recently, we showed that PD‐1 can also be expressed by bystander T cells in the tumors,^2^ indicating that this receptor is not exclusively expressed by tumor‐specific cells, and raising questions about the effects of anti‐PD‐1 treatment on these cells. Surprisingly, the two populations of EBV‐specific CD8^+^ TILs infiltrating this EBV‐driven LELC tumor only partially expressed PD‐1, 13.8% and 52.6% of tetramer^+^ cells, respectively, for BMLF1 and BRLF1 epitopes (Figure 2e). Furthermore, the intensity of PD‐1 expression on these cells was lower than that of PD‐1 expression on tumor‐associated antigen or neoantigen‐specific CD8^+^ TILs (Supplementary figure 1b). Nonetheless, we noted that the tumor cells expressed high level of PD‐L1 in this tumor (Figure 2f). Because of the low and partial PD‐1 expression on EBV‐specific CD8^+^ TILs infiltrating EBV‐driven LELC, our data suggested that anti‐PD‐1 treatment for EBV‐driven LELC may not be able to disinhibit these EBV‐ and tumor‐specific cells.

## Discussion

In this study, we report the identification of two EBV‐specific CD8^+^ T‐cell populations infiltrating an EBV‐driven LELC of the lung. Tumor‐specific CD8^+^ cells have been characterised by an exhausted/dysfunctional signature, which can be reverted by immunotherapy.^26^ However, CD8^+^ TILs are highly heterogeneous across tumors, patients and cancer types.^2^, ^26^ This heterogeneity across patients can be explained by tumor cell‐intrinsic factors shaping the tumor immune microenvironment and finally influencing the outcome of immunotherapy.^27^ Recently, we demonstrated that intra‐tumor heterogeneity can be in part explained by the presence of both tumor‐specific (exhausted phenotype, CD39^+^) and bystander CD8^+^ TILs.^2^ Here, within the same tumor microenvironment, we report distinct exhaustion signatures between two tumor‐specific CD8^+^ TIL populations. Furthermore, each of these two tumor‐specific populations is phenotypically distinct. We previously reported similar observations in a mouse tumor model.^28^ This heterogeneity within the same tumor environment suggests that unidentified factors (e.g. tumor‐epitope availability, spatial localisation or TCR repertoire diversity/uniformity) could influence the acquisition of different exhausted/dysfunctional profiles. As reported previously, LELC tumor cells express high level of PD‐L1,^29^ making LELC cancer a potential good candidate for anti‐PD‐1 or anti‐PD‐L1 immunotherapy. However, case report showed variable effects of these treatments in LELC, with partial^30^ or no response.^31^ Surprisingly, we observed in our LELC patient that both tumor‐specific CD8^+^ TILs only partially expressed PD‐1 (13.8% and 52.6%). This observation could explain the partial absence of response of LELC patients to anti‐PD‐1 treatment. This observation strongly suggests that targeting PD‐1–PD‐L1 pathway by immunotherapy in LELC might not be the most appropriate strategy. Of note, relatively low rates of response to anti‐PD1 immunotherapy have been observed for NPC as well, which is also an EBV‐associated carcinoma.^32^, ^33^ It is interesting that EBV‐related tumors do not show high response rates to checkpoint blockade immunotherapy despite the strong T‐cell immunogenicity of EBV. Our data showing that tumor‐infiltrating EBV‐specific cells can display hallmarks of chronic antigen stimulation and exhaustion even without PD‐1 expression may help to explain this. Additional studies will be needed to investigate the benefits of immunotherapy in EBV‐driven malignancies based on other epitopes or HLA responses. Knowing that the expression of PD‐1 on EBV‐specific T cells can be highly heterogeneous across patients and depends on the state of differentiation,^34^ personalised immunotherapy could be a more suitable strategy.

Effective treatment options for LELC are limited. The oncogenic role of EBV has been attributed to the expression of latent genes providing growth and survival benefit to DNA‐damaged epithelial cells. Several therapeutic strategies have been developed to induce a cytotoxic CD8^+^ T‐cell (CTL) response targeting specifically the latent protein, such as autologous CTL infusion,^35^ vaccination with EBV latent vector^36^ or pulsed‐dendritic cells.^37^, ^38^ However, these strategies show moderate efficacy, with toxicity in some cases.^39^ In our study, we only detected EBV‐specific CD8^+^ TILs for lytic proteins (BMLF1 and BRLF1). The oncogenic role of EBV has been attributed to the expression of latent genes providing growth and survival benefit to DNA‐damaged epithelial cells.^40^ However, recent data highlighted that EBV lytic genes are detected in EBV‐associated malignancies as well.^41^, ^42^, ^43^ Moreover, recent works suggest that EBV lytic cycle can contribute to carcinogenesis through the induction of oncogenic cytokine secretion and genome instability.^44^, ^45^ In NPC, recurrent induction of EBV lytic cycle contributes more profoundly to NPC carcinogenesis.^46^ Taken together, our observations suggest that targeting lytic proteins combined with latent proteins could be more efficient for autologous CTL infusion or vaccination strategies. In the last few years, chimeric antigen receptor (CAR) T‐cell therapy has shown promising efficacy in the treatment of B lymphoma.^47^, ^48^ Similarly, TCR‐engineered T cells targeting tumor epitopes have been developed and are used in phase I trials (i.e. MAGE3, NY‐ESO).^49^ Engineered T cells expressing a TCR targeting the latent EBV protein LMP1 presented by HLA‐A*02: 01 have been generated in a preclinical model.^50^ Based on our observations, targeting lytic EBV epitopes presented by tumor cells could present an interesting alternative strategy in EBV‐driven cancer, because of the presence of the same EBV epitopes in different patients and their absence in non‐infected normal cells. However, our data highlight the polyclonal TCR repertoire of EBV‐specific CD8^+^ TILs as well. Of note, because of the low frequency of the TCR transcript, our approach could lead to a bias in clonotype hierarchy. Thus, more studies are needed to (1) evaluate the efficiency of engineered T cells targeting lytic vs. latency‐associated EBV proteins and (2) evaluate the efficiency of monoclonal versus polyclonal TCR‐engineered T cells. Overall, our investigation of TIL specificities in the context of a virus‐associated cancer should help improve and design new targets for future immunotherapeutic modalities.

## Methods

### Human samples, Immunohistochemistry and cell isolation

PBMC and tumor samples were obtained from a 69‐year‐old Asian woman, non‐smoker, with a lung cancer. Immunohistochemistry for H&E, PD‐L1 and EBERish was performed on PFA‐fixed tissue sections.^51^ Tumor single‐cell suspensions were prepared as previously described.^52^ Briefly, tissues were mechanically dissociated in small pieces and incubated at 37ºC for 15 min in DMEM + collagenase IV (1 mg mL^−1^) + DNase (15 µg mL^−1^). Digestion was stopped by the addition of RPMI + 5% FBS. Dissociated tissues were filtered and washed in RPMI + 5% FBS + DNase (15 µg mL^−1^). Single‐cell suspensions were cryopreserved in 90% FBS + 10% DMSO and stored in liquid nitrogen. The use of human tissues was approved by the appropriate IRBs, A*STAR, the Singapore Immunology Network.

### HLA typing and MHC class I tetramer staining

HLA typing was performed by sequence‐specific primer PCR as previously described.^53^ HLA‐A*24: 02 monomer with a UV cleavable peptide was produced as described previously.^54^ Peptide exchange was performed using the following peptides specific for EBV: LMP2 (HLA‐A*24: 02 – IYVLVMLVL), EBNA3 (HLA‐A*24: 02 – RYSIFFDYM), BRLF1 (HLA‐A*24: 02 – TYPVLEEMF), BMLF1 (HLA‐A*24: 02 – DYNFVKQLF) and EBNA3B (HLA‐A*24: 02 – TYSAGIVQI). Each MHC class I monomer was tetramerised using streptavidin at the ratio 4: 1 as described previously.^55^ Frozen samples were thawed and washed. Cells were incubated for 1 h at room temperature with the tetramer. Cells were then incubated with an antibody cocktail for 15 min and acquired on flow cytometry.

### Mass‐cytometry experiment

Purified antibodies lacking carrier proteins were conjugated to a heavy metal according to the protocol provided by Fluidigm Inc. (San Diego, CA, USA) Streptavidins were heavy metal‐labelled as previously described.^54^ Frozen samples were thawed and washed in RPMI + 10% FBS + DNase (15 µg mL^−1^). Cells were incubated for 1 h at room temperature with the tetramer cocktail. Then, cells were stained with cisplatin (viability marker) at 5 µM in PBS for 5 min at 4°C. Cells were then incubated with an antibody cocktail for 15 min and fixed in PFA 2% prior to CyTOF acquisition.

### mRNA sequencing data analysis

The paired‐end RNA‐seq reads from HiSeq 4000 were mapped to Human GRCh38/HG19 reference genome using STAR software tool. The mapped paired‐end reads were summarised to gene level using featureCounts v1.5.0‐p1 software tool and with GENCODE v26 gene annotation. Genes with read count less than 5 in less than 2 samples in all cell populations were filtered out from further analysis. Limma‐voom pipeline was used for differentially expressed gene (DEG) analysis. DEGs from comparisons between different cell populations were selected with the Benjamini–Hochberg adjusted *P*‐value of < 0.05. All analyses were done in R‐3.1.2. We used the *HTSanalyzeR* package (v 2.26.0) to run GSEA on gene collections from the T‐cell exhaustion gene set^56^, ^57^ filtered for gene sets with at least 20 genes present in our data set. For GSEA, we used 1000 permutations to estimate *P*‐values and applied corrections for multiple tests using the Benjamini–Hochberg procedure. To measure TCR diversity, we converted the measured counts for each TCRα and TCRβ to frequencies.

### Data analysis and UMAP

After CyTOF acquisition, which was performed as previously described,^58^ any zero values were randomised using a uniform distribution of values between zero and minus one using a R script. Note also that all other integer values measured by the mass cytometer are randomised in a similar fashion by default. The signal of each parameter was then normalised based on the EQ beads (Fluidigm). Samples were then used for UMAP analysis similar to that previously described.^21^ In R, all data were transformed using the 'logicleTransform' function ('flowCore' package) using the parameters *w* = 0.25, *t* = 16409, *m* = 4.5 and *a* = 0 to roughly match scaling historically used in FlowJo.

## Conflict of interest

Evan W Newell is a co‐founder, advisor and shareholder of ImmunoScape Pte Ltd and an advisor for Neogene Therapeutics and NanoString Technologies.

## Author contributions

Yannick Simoni: Design of experiments; conduction of experiments; data analysis; manuscript writing. Shamin Li: Data analysis; review of manuscript. Etienne Becht: Analysis of transcriptomic data. Chiew Yee Loh: Sample processing. Joe Poh Sheng YEONG, Tony Kiat Hon Lim, Angela Takano and Daniel Shao Weng Tan: Provision of samples; discussion of data. Evan W Newell: Project initiation; led project; development of scripts for CyTOF analysis; manuscript writing.

## Supporting information

Supplementary figure 1
