# Supplementary material for: Partial absence of PD‐1 expression by tumor‐infiltrating EBV‐specific CD8+ T cells in EBV‐driven lymphoepithelioma‐like carcinoma
Source: Clin Transl Immunology. 2020 Sep 9;9(9):e1175. doi: 10.1002/cti2.1175 (PMC7503213; doi:10.1002/cti2.1175)
Supplement: Supplementary file 1 — Supplementary figure 1 [file CTI2-9-e1175-s001.pdf]

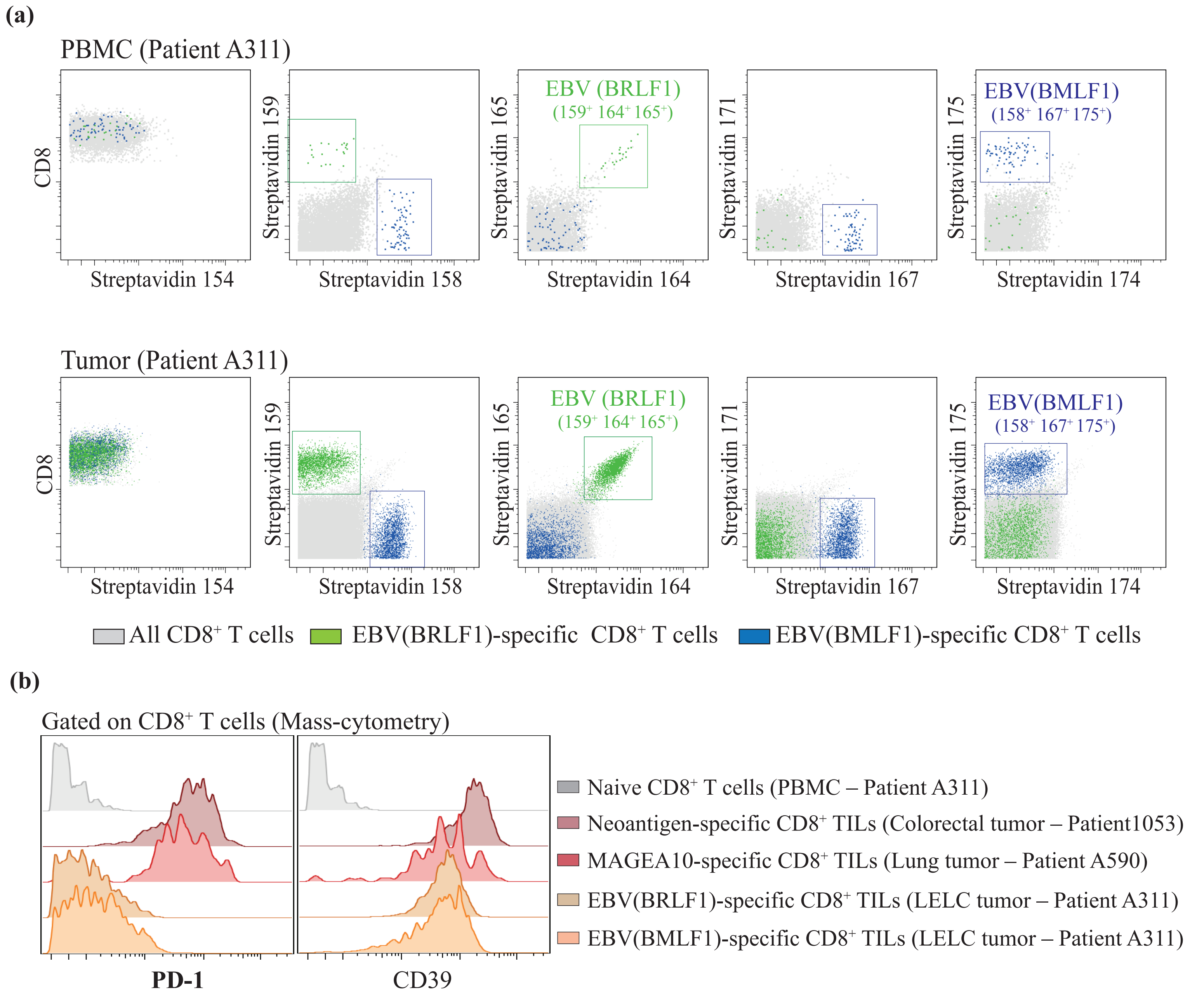

Supplementary figure 1. Identification and characterization of EBV-specific CD8$^+$ TILs in EBV-driven LELC tumor by mass cytometry. **(a)** Multiplex tetramer staining by mass cytometry. To investigate the antigen specificity of CD8$^+$ T cells in blood and tumor of LELC patient (Patient A311), we performed multiplex MHC class I tetramer staining as reported previously (see Methods). By using a three-metal coding scheme, we encoded up to 120 different tetramers specific for viral epitopes and tumor-associated antigens. EBV(BMLF1)-specific CD8$^+$ T cells were identified using HLA-A*24:02 tetramer (DYNFVKQLF), EBV (BRLF1)-specific CD8$^+$ T cells were identified using HLA-A*24:02 tetramer (TYPVLEEMF). **(b)** Expression of PD-1 and CD39 by EBV-, tumor associated antigen- and neoantigen-specific CD8$^+$ TILs in human cancer. Histogram representing PD-1 and CD39 expression by tumor-specific CD8$^+$ TILs identified by MHC class I tetramer. Naive CD8$^+$ T cells (CCR7$^+$ CD45RO$^-$), Neoantigen-specific CD8$^+$ TILs were identified using HLA-A*11:01 tetramer for mutAHR (GISQELPYK) (see Simoni *et al.* Nature 2018), tumor associated antigens MAGEA10-specific CD8$^+$ TILs were identified using HLA-A*02:01 tetramer (GLYDGMEHL).
